# LONGITUDINAL HEALTH CONSEQUENCES OF CHILDHOOD ADVERSITY: THE MEDIATING ROLE OF PURPOSE IN LIFE

**DOI:** 10.1093/geroni/igz038.223

**Published:** 2019-11-08

**Authors:** Kristin J Homan

**Affiliations:** Grove City College, Grove City, Pennsylvania, United States

## Abstract

Adverse childhood experiences have long-term detrimental effects on physical health. Although biological, behavioral, and social factors have been explored as intermediate mechanisms, little research has explored psychosocial factors as potential mediators. This study examined whether purpose in life longitudinally mediates the relationship between childhood adversity and two measures of adult health (self-rated health and functional limitations). Data were obtained from 3,871 participants in the Midlife in the United States (MIDUS) study. We tested a cross-lagged mediation model from childhood adversity to adult health via purpose in life, controlling for baseline measures of health and purpose in life. Good model fit was achieved indicating that childhood adversity is associated with poorer adult health through direct and mediated paths. Childhood adversity may restrict young people’s search for purpose in life, and reduced purpose in life is ultimately associated with poorer subjective health and increased functional limitations.

